# The Interplay among Age and Employment Status on the Perceptions of Psychosocial Risk Factors at Work

**DOI:** 10.3390/ijerph17103611

**Published:** 2020-05-21

**Authors:** Valerio Ghezzi, Tahira M. Probst, Laura Petitta, Valeria Ciampa, Matteo Ronchetti, Cristina Di Tecco, Sergio Iavicoli, Claudio Barbaranelli

**Affiliations:** 1 Department of Psychology, Sapienza—University of Rome, 00146 Rome, Italy; laura.petitta@uniroma1.it (L.P.); valeria.ciampa@uniroma1.it (V.C.); claudio.barbaranelli@uniroma1.it (C.B.); 2 Department of Psychology, Washington State University, Vancouver, WA 98686, USA; probst@wsu.edu; 3 Department of Occupational and Environmental Medicine, Epidemiology and Hygiene, INAIL—Italian Workers’ Compensation Authority (INAIL), 00078 Monte Porzio Catone (Rome), Italy; m.ronchetti@inail.it (M.R.); c.ditecco@inail.it (C.D.T.); s.iavicoli@inail.it (S.I.)

**Keywords:** age, aging, Bayes factor, contingent work, employment status, psychosocial risk factors at work, Bayesian informative hypotheses, work-related stress, work contract

## Abstract

While the role of individual differences in shaping primary appraisals of psychosocial working conditions has been well investigated, less is known about how objective characteristics of the employee profile (e.g., age) are associated with different perceptions of psychosocial risk factors. Moreover, previous research on the link between employment status (i.e., work contract) and such perceptions has provided mixed results, leading to contradictory conclusions. The present study was conducted on a nationally representative sample of theItalian employed workforce surveyed with computer-assisted telephone interviewing (CATI) methodology. The principal aim of the study is to bridge this gap in the extant literature by investigating the interplay between two key characteristics of the employee profile (i.e., age and work contract) in shaping employees’ perceptions of psychosocial risk factors. Given the disparate literature scenario on the interplay between age and employment status in shaping primary appraisals of psychosocial stressors, we formulated and compared multiple competitive informative hypotheses. Consistent with the principles of the conservation of resources (COR) theory, we found that older contingent employees reported a higher level of psychosocial risk than their permanent peers who, in turn, were more vulnerable than middle-aged and younger workers (regardless of their employment status). These results highlight the importance of simultaneously assessing multipleobjective variables of the employee profile (i.e., age and employment status) which may act to shape subjective perceptions of psychosocial risk factors for work-related stress. Given our findings, employers and policy makers should consider older contingent employees as one of the workforce sub-populationsmost vulnerable to negative work environments.

## 1. Introduction

Modern organizations are multifaceted systems where multiple variables from different sources contribute to determining employee well-being and optimal performance. Consistent with the increasing complexity of job design processes [1,2] and the growing flexibility of the labor market [3,4], employees are constantly exposed to multiple job demands, namely those “physical, psychological, social, or organizational aspects of the job that require sustained physical and/or psychological (i.e., cognitive or emotional) effort and are therefore associated with certain physiological and/or psychological costs” [5] (p. 296). Not surprisingly, there is evidence that excessive job demands may turn into job stressors when key employee resources are lost or when they are under persistent threat of loss [6]. If not mitigated by adequate personal and/or organizational resources, job stressors are likely to produce serious consequences for employee well-being [7], and their effects are likely to translate into work-related stress [8]. Notably, work-related stress may seriously impair employees’ psychological and physical health [9,10,11,12,13] as well as their job performance [14,15,16], producing impressive costs for healthcare and welfare systems.

Although work-related stress is determined by multiple causes [17], none hasbeen as extensively studied as psychosocial risk factors, namely those features of work design concerned with the overall organizational context which may impair the psychological and/or physical well-being of the employees [18]. Such risk factors may be meaningfully conceptualized as job demands associated with different working conditions [19], such as task demands, control, role conflict, and lack of social support [20]. While the role of individual differences in the subjective evaluation of psychosocial stressors has been extensively investigated (e.g., personality and coping strategies, see [21]), less is known about the role played by objective employee characteristics in shaping the subjective appraisal of psychosocial risk factors [22,23,24]. For example, the conservation of resources (COR) theory [6] posits that losses in resources may contribute to exposing employees to a higher risk of work-related stress, and this includes objective resources as well. Thus, using a statistically representative sample of the Italian employed workforce, the present study examines the interplay between two pivotal objective elements of the employee profile (i.e., age and employment status) in relation to perceptions of psychosocial risk factors.

### 1.1. Age and Psychosocial Risk Factors at Work

Rauschenbach and Hertel [25] proposed three possible patterns of differences among older and younger employees in relation to the subjective evaluation of psychosocial stressors. The first hypothesis frames older employees as less vulnerable to psychosocial stressors than others. As argued by Hertel, Rauschenbach, Thielgen, and Krumm [26], problem-based and emotion-based coping strategies closely resemble, respectively, primary and secondary control mechanisms posited by the lifespan theory [27]. On the one hand, older employees use secondary control (i.e., emotion-focused coping mechanisms) more efficiently and effectively to face job demands [28,29] since the frequency of the adoption of these strategies increases with age [30]. On the other hand, the effectiveness of primary control (i.e., problem-focused coping mechanisms) is generally stable over time and the frequency of its use increases at a younger age, peaks during midlife (e.g., between 45 and 65 years) and stabilizes until retirement from work [31]. Given the substantial stability of problem-focused coping strategies across the lifespan and the advantage of older employees in emotion-based coping strategies over their younger counterparts, the former group of employees should perceive psychosocial demands as less threatening than the latter.

In this regard, the selection, optimization andcompensation (SOC) model [32] posits that people may achieve successful aging by selecting only the goals they assess as more valuable (S), and they may optimize resources aimed at achieving them (O). Moreover, they counteract unavoidable losses in relevant resources (e.g., cognitive and physical) by investing other available resources in different activities (C). For example, as noted by Rauschenbach, Krumm, Thielgen, and Hertel [33], older workers “might successfully apply selection strategies during the work-life cycle in order to minimize the occurrence of stressors” (p. 784).

From an empirical standpoint, several studies support this hypothesis. Hertel et al. [26] found in their longitudinal study that age was positively related to a higher probability to adopt problem-focused coping strategies at work, which in turn reduced stress after an eight-month lag. In contrast, age was negatively related to avoidance-based coping strategies under conditions of low job control. Scheibe, Spieler, and Kuba [34] found that older employees were more prone to use adaptive emotion-based regulation strategies (e.g., positive reappraisal) while younger ones adopt more frequently maladaptive emotion-based regulation strategies (e.g., rumination). Such findings were also consistent with other studies (e.g., [35]) and with the conclusions outlined in a recent systematic review [28], which argued that older individuals may be considered the best “copers” from an emotional regulation perspective, which also applies to the workplace and organizational contexts [29]. Finally, Mauno, Ruokolainen, and Kinnunen [36] showed that older employees handle the negative effects of workload and work–life imbalance on job satisfaction more efficiently than younger employees.

A second hypothesis outlined by Rauschenbach and Hertel [25] posits that older employees may experience their working conditions as more stressful than their younger colleagues because of their higher vulnerability to job demands and their fewer resources to counterbalance them (e.g., cognitive and physical resources, see [37]). For instance, fluid intelligence (e.g., processing new information) substantially declines with age [38] and older workers are less equipped to handle multiple tasks [39]. At the same time, physical abilities also decline with age [40], and general work abilities are reduced [41,42]. As suggested by Rauschenbach et al. [33], the SOC model [32] may also be used as a theoretical platform to support the hypothesis of higher vulnerability to psychosocial stressors for older employees. Specifically, there may be relevant barriers for older workers to select appropriate strategies to compensate for resource loss when multiple demands occur simultaneously in the workplace [43]. Moreover, the actual adoption of SOC strategies was found to be very weakly associated to age [44]. In this sense, despite the solid platform of experiences for the effective use of personal resources to offset psychosocial risks that older employees may have acquired, the resources they have lost may be more salient for stress processes than those resources they gathered throughout their career [45].

In support of this hypothesis, Yaldiz, Truxillo, Bodner, and Hammer [46] found that the effect of poor job resources on work-related stress is higher for older than younger workers, while Kiss, De Meester, and Braeckman [47] showed that older employees may take longer to recover from high demands in a large sample of employees from the public sector. Further, Mayes, Barton, and Ganster [48] found that the impact of role conflict on strain was stronger for older employees, and similar results were found in the Italian workforce by Marinaccio and colleagues [22]. Other findings show a stronger association among work demands and tension for older employees and also a higher propensity to experience job changes as more hindering than younger employees [49]. Finally, Marinaccio and colleagues [22] found that older shift and/or night workers displayed poorer performance than others in several psychosocial risk factors (i.e., task demands, lack of control, managerial and peer support, and support for change).

The third hypothesis about the relationship between age and the appraisal of psychosocial risks consists of an inverted U-shaped relationship, where middle-aged employees may be more susceptible to the negative effects of psychosocial job demands than their younger and older colleagues. Heckhausen [50] described middle age as a ‘sandwich’ life stage “between a life period with a predominant growth orientation and a life period that is confronted with many losses and declines” [25] (p. e51), where individuals are invested in multiple responsibilities and duties within and outside the workplace (e.g., family). As with the previous two hypotheses, this hypothesis has also received some empirical support. For example, Aldwin, Sutton, and Lachman [51] found in a sample of men that middle-aged individuals more frequently reported stressful episodes than other age groups and they appraised such problems as challenging or annoying. Similarly, Rauschenbach and Hertel [25] found a quadratic effect of age on reported strain and negative affect experienced at work. This curvilinear relationship has been also observed in relation to several aspects of employee well-being (for a review, see [52]).

Overall, these perspectives addressing the relationship between age and psychosocial risks offer a very fragmented scenario (on this point, see also [33]), highlighting important inconsistencies among theoretical models and empirical findings. For these reasons, the three alternative hypotheses outlined by Rauschenbach and Hertel [25] will serve as a platform to formulate theoretically sound competitive hypotheses in the following sections of the present work.

### 1.2. Employment Status and Psychosocial Risk Factors at Work

The use of non-standard contractual forms has rapidly increased during the last three decades all over the world [53], especially for young workers (e.g., [54]). As illustrated by De Witte and Näswall [55], researchers often refer to the absence of permanent contracts with a plethora of labels capturing some distinctive aspects of non-permanent employment status (e.g., non-standard work, temporary, agency, fixed-term contracts, etc.). For this study, we refer to contingent status as the employment condition characterized by the absence of open-ended contractual arrangements. Pearce considered the non-permanent job contract as an “objective” form of job insecurity, which is mainly characterized by “an independently determined probability that workers will have the same job in the foreseeable future” [56] (p. 34). As such, contingent employment status has been frequently studied as a psychosocial risk per se [57] or as an objective proxy of subjective job insecurity (e.g., [58]). Compared to permanent employees, individuals with a contingent employment status often report poorer physical health [59,60], poorer psychological adjustment [61,62], poorer health and safety at work [63,64], and they typically experience greaterrole ambiguity under poorer working conditions [65].

From the perspective of work-related stress theory, contingent employees are generally exposed to three distinct categories of stressors (see [66]) above and beyond those related to working conditions shared with their permanent colleagues. First, employers are likely to consider the contingent workforce as “peripheral” to the organization, resulting in a lack of significant investments into contingent employees in terms of training opportunities, wages, and promotions (e.g., [67]). Second, contingent employees may be more vulnerable to work-related stress because they have “reduced control, role stress and limited support” [66] (p. 29) compared to their permanent counterparts in the workforce. For example, contingent employment status may be associated with lower job autonomy (e.g., [68]), lower skills development, and less job responsibilities [69], as well as less support by their permanent colleagues [70]. Moreover, it is quite common that contingent employees are exposed to stereotypical judgments related to their employment status (e.g., [71]). Third, contingent workers are uniquely exposed to so-called “employment strain” [72,73], which is characterized by high demands coupled with low control over the employment relationships (rather than over the job), such as the continuous need to search and maintain a job, expectations towards contract renewal, and uncertainty surrounding compatibility of one’s actual skills to the available job positions in the labor market. In this vein, Marinaccio and colleagues [22] showed that contingent employees may perceive less role clarity than their permanent colleagues. Finally, De Sio and colleagues found significant differences between permanent (less vulnerable) and non-permanent (more vulnerable) administrative technical workers in all psychosocial risk factors assessed via the Health and Safety Executive Management Standards Indicator Tool [74].

Taken together, available findings depict contingent employees as more vulnerable to psychosocial risks than permanent ones.

### 1.3. Framing Age and Employment Contingent Status within COR Theory

The first corollary of the COR theory proposes that individuals “who lack resources are more vulnerable to resource loss and less capable to resource gain” [6] (p. 104) and, in turn, individuals with a more consistent baseline of resources suffer less from such losses; further, they are more prone to gain resources. Since both employee aging and contingent status are objective conditions of lack of resources and threats to resource loss, it is likely that both older and contingent employees will be more vulnerable to the risk of work-related stress than their younger and permanent employee counterparts who, in turn, are more likely to gain resources.

The second corollary of the COR theory (i.e., resource loss cycles) proposes that at each iteration of resource loss (or threatened loss) individuals will have less resources to offset the negative effects of new resource loss. This spiraling effect is also posited to increase in magnitude with each cycle. In line with this COR theory prediction, it is likely that negative effects of aging and contingent status may accumulate and make it even more difficult to gain new resources to counteract such an unavoidable (and mutual) process of exacerbation in resource loss. Consistent with this, such resource loss cycles may lead older contingent workers to perceive psychosocial risk factors more negatively than other employees.

Finally, COR theory posits three basic conditions for the onset of work-related stress, which may occur: “(a) when central or key resources are threatened with loss, (b) when central or key resources are lost, or (c) when there is a failure to gain central or key resources following significant effort” [6] (p. 104). Consistent with these principles, we can assume that older age (which increases the likelihood to key resource loss and depletion) and contingent employment (which reflects an “objective” threat of resource loss) may play an important role in shaping the perceptions of psychosocial risk factors, and their interplay may hinder new resource gains to counterbalance the detrimental effects of such objective employee characteristics.

### 1.4. The Present Study

Given these premises and our theorizing above, the aim of the present study was to evaluate the conjoint effect of age and employment status on the perceptions of psychosocial risk factors at work in a large representative sample of Italian workers. Specifically, this study aimsat translating into testable and comparable hypotheses the available knowledge regarding the interplay among anemployee’s age and his/her employment status in shaping primary appraisals of psychosocial risk factors at work. 

Although we provide specific predictions in line with the principles of the COR theory, we detailed in previous sections a very inconsistent pattern of findings concerning the relationship between age and such psychosocial risk factors. Accordingly, and drawing on prior knowledge discussed above, we formulate a series of competitive inequality constrained informative hypotheses [74] in order to contrast our specific expectations with alternative theoretically and methodologically sound models concerning the interplay of age and employment status in shaping perceptions of psychosocial risk factors.

#### Defining a Set of Plausible Informative Hypotheses

Inequality constrained informative hypotheses (for a comprehensive overview, see [75]) allow for directly testing research expectations and contrasting them with other substantively grounded hypotheses. As noted by Kluytmans, Van De Schoot, and Hoijtink [76], there are several advantages in adopting an informative hypothesis perspective to test research expectations over the classical “frequentist” approach. First, researchers can formally and directly express their substantive hypotheses by using (in)equality constraints and testing to what extent they are supported from the data. Second, prior knowledge is largely emphasized for the development of substantive hypotheses and their potential “competitors” (e.g., a pool of alternative hypotheses) while the classical null hypothesis significance testing (NHST) framework (see [77]) only allows for testing (bidirectionally or monodirectionally) one’s expectations against the null hypothesis (i.e., presence of an effect vs. no effect). Third, the informative hypothesis approach avoids the arbitrary use of *p*-values (see [78]) to determine statistical significance of an effect and overcomes the limitations of power loss in multiple comparisons (e.g., Bonferroni correction for multiple comparisons). Fourth, results are easier to report and to communicate to different stakeholders.

As the first informative hypothesis of our pool, we posit that age and employment contract might not have any effect on the perceptions of psychosocial risks. This is the Bayesian version of the classical “null” hypothesis of one-way ANOVA models evaluated with omnibus tests within the NHST approach [74], and it generally serves as a benchmark for other substantive competitive hypotheses in their final (Bayesian) comparative evaluation. Thus, we formally express that:

H_0_: μY*_p_* = μY*_c_* = μM*_p_* = μM*_c_* = μO*_p_* = μO*_c_*; (1)

where μ stands for the average level of psychosocial risks within a given age*employment status group; Y, M, and O stand, respectively, for younger, middle-aged, and older employees;and *p* and *c* subscripts stand for permanent and contingent employment conditions, respectively.

A second set of informative hypotheses concerns the primary role of age in shaping the perceptions of psychosocial risks. As illustrated in previous sections, Rauschenbach and Hertel [25] proposed three different forms of such relationships (i.e., each group of older, middle-aged, and younger employees may perceive more negatively psychosocial risk factors than other groups). Since each hypothesis received consistent empirical support, we can formally translate such prior knowledge into the following informative hypotheses:

H_1_: μO*_p_* = μO*_c_* > μM*_p_* = μM*_c_* = μY*_p_* = μY*_c_*; (2)

H_2_: μM*_p_* = μM*_c_* > μY*_p_* = μY*_c_* = μO*_p_* = μO*_c_*; (3)

H_3_: μY*_p_* = μY*_c_* > μM*_p_* = μM*_c_* = μO*_p_* = μO*_c_*; (4)

For example, in H_1_ we put a single inequality constraint among the means of older contingent and middle-aged permanent age groups, so that we are testing directly that older employees are more at risk than all of the other employees, hypothesizing no differences among the older contingent and older permanent employees.

A further set of informative hypotheses concerns the primary role of contingent employment in shaping perceptions of psychosocial risk factors. Specifically, being a contingent employee may increase the likelihood of having high psychosocial risk factors per se, regardless of the employee age. This hypothesis is consistent with the view that contingent employees are exposed to a larger number of stressors and with a higher intensity than permanent workers (e.g., [66]). Thus, we formally express that:

H_4_: μY*_c_* = μM*_c_* = μO*_c_* > μY*_p_* = μM*_p_* = μO*_p_*; (5)

However, the opposite situation is also likely to occur under some circumstances. For example, in their longitudinal study, Parker, Griffin, Sprigg, and Wall [79] found that temporary workers experienced lower strain-induced demands (e.g., role overload) than permanent workers. Although these findings have received little additional support in the literature, we nevertheless translate them into a further competitive informative hypothesis. In other words, one might expect that permanent workers experience psychosocial stressors with greater intensity than their contingent colleagues, which can be formally expressed as follows:

H_5_: μY*_p_* = μM*_p_* = μO*_p_* > μY*_c_* = μM*_c_* = μO*_c_*; (6)

The next set of informative hypotheses is concerned with the potential accumulative effect of age and contingent employment on the perceptions of psychosocial risks. As noted above, there is evidence that older, middle-aged, and younger employees may be, for different reasons, more at risk than others regarding psychosocial factors [25,33]. In the following informative hypotheses, we add the role of contingent employment status in exacerbating such perceptions for each specific age class. These hypotheses are consistent with the COR principle of the resource loss cycle [6], where individuals lacking in important resources have difficulties in facing new threats of resource loss. Accordingly, since being an older, middle-aged or younger worker can all constitute potential conditions where some relevant resources are lacking to counteract workplace stressors, also being in a contingent employment status may increase the likelihood of higher vulnerability to psychosocial factors. At the same time, being a permanent employee under conditions of resource loss (i.e., older, middle or younger age) may serve as a compensatory resource hindering negative perceptions of the work environment. Thus, we also formulate that:

H_6_: μO*_c_* > μO*_p_* = μM*_p_* = μM*_c_* = μY*_p_* = μY*_c_*; (7)

H_7_: μM*_c_* > μM*_p_* = μY*_p_* = μY*_c_* = μO*_p_* = μO*_c_*; (8)

H_8_: μY*_c_* > μY*_p_* = μM*_p_* = μM*_c_* = μO*_p_* = μO*_c_*; (9)

Finally, we add to the previous set of hypotheses an additional inequality constraint between the permanent workers of a given age class and the other employees. In this case, having a permanent employment status within a given age period may only partially hamper the negative perceptions of psychosocial risks at work. The following hypotheses are fully consistent with the COR principles (see [6]). Specifically, they consider both the loss in key resources associated with being a given age (e.g., older age) and the accumulative effect of being a contingent employee with that age as a threat for additional resource loss (i.e., resource loss cycles); in addition, being permanent only partially attenuates the negative experience of psychosocial risks within the workplace because resource loss (associated with age) is more salient than resource conservation and gains (associated, in this case, with the permanent employment status). Thus, we hypothesized different gradients of the interplay among age and employment status in shaping the perceptions of psychosocial risks. Taking H_9_ as an exemplar, we posited that older contingent workers may be more at risk than older permanent workers who, in turn, may be more at risk than other employees.

H_9_: μO*_c_* > μO*_p_* > μM*_p_* = μM*_c_* = μY*_p_* = μY*_c_*; (10)

H_10_: μM*_c_* > μM*_p_* > μY*_p_* = μY*_c_* = μO*_p_* = μO*_c_*; (11)

H_11_: μY*_c_* > μY*_p_* > μM*_p_* = μM*_c_* = μO*_p_* = μO*_c_*; (12)

Despite proposing several competitive informative hypotheses about the relationship and interplay of our focal objective variables with psychosocial risks, we expect that being an older employee with a contingent employment status exposes individuals to a more pronounced negative experience of working conditions, followed by older permanents which, in turn, may be more at risk than other employees. In essence, we expect that the data will support H_9_ to a greater extent than the alternative plausible informative hypotheses.

## 2. Materials and Methods

### 2.1. Procedure

This study was based on data from the INSuLa (*Indagine Nazionale sulla Salute e Sicurezza sul Lavoro*) project, the principal cross-sectional survey representative of the Italian work population conducted in 2013 by the Italian Workers’ Compensation Authority [80]. The survey was aimed at investigating workers’ perceptions of health and safety at work. The reference statistical population comprised the Italian national workforce aged from 16 to 64 years excluding self-employed, military, and civil protection personnel. The National Labor Force Survey conducted in 2012 provided information to define the statistical universe and to stratify the sample of employees based on their region, gender, age, type of contract, occupational level, and occupational sector, in collaboration with the Italian National Institute of Statistics (ISTAT). Specifically, the target population wasthe Italian employees who worked at least an hour a week for at least two continuous months in the past 6 months, and whose employment was regulated by the national legal framework for health and safety (Legislative Decree 81/08). Therefore, all professionals with specific regulations (such as the armed forces or civil protection volunteers) and self-employed workers and entrepreneurs were excluded. Since there was a lack of official national data, ISTAT extracted representative data of around 17,000 employees from their last national workforce survey. This information was used to define the strata and their quota for the socio-demographic and occupational characteristics mentioned above. Eligible subjects were previously identified using a random procedure and contacted via telephone (mobile and landline). Participants were selected proportionally based on the sampling strategy described above in order to match the population characteristics, and they endorsed an informed consent read by the interviewer before they were surveyed.

Data were collected from July to December 2013 through structured interviews conducted by trained interviewers using the computer-assisted telephone interviewing (CATI) methodology. The data collection was conducted in collaboration with TNS Italia, an institution experienced in social research surveys.

Previously the survey questionnaire was tested through a pilot study to check its compatibility with the CATI methodology in regard to the duration of the interview and clarity of the questions; specifically, 50 interviews were conducted during the pilot study.The study was conducted in accordance with the Declaration of Helsinki and its further modifications.

### 2.2. Participants

The initial sample comprised 8000 employees (54% males) with a mean age of 42.97 years (range 19–64, SD = 9.77). Table 1 shows the socio-demographic and occupational characteristics of the sample.

The large majority of the sample comprised Italian natives (97.2%) while the remaining participants were from other countries but currently employed in Italy and speaking fluent Italian. 

Participants were mainly employed in organizations from the private (65.6%) and public sectors (28.5%); organizational size ranged from <10 (15.6%), to 50 (19.6%), to 250 (21.4%), and 250+ (39.2%) and missing (4.2%)

Job tenure (i.e., the length of time an employee has worked for their employer) was higher than ten years for more than 83% of the sample, while organizational tenure was higher than five years for more than the 72% of the sample.

Finally, participants were employed in different economic and industrial sectors: manufacturing/industry/energy (23.2%), wholesale and retail trade, hotels, and restaurants (17.7%), and education and public administration (14.9%) were those most frequently represented by the data.

The majority (85%) of the sample had a permanent contract, while 14.5% had different forms of contingent employment contract (i.e., fixed-term, apprenticeship, project contracts, seasonal work, temporary, and agency work). A small percentage of employees (0.5%) did not provide this information, and for this reason they were dropped from the final sample *ofn* = 7957 employees

Finally, sampling weights were derived in order to restore appropriate proportions among the different sampling strata and provide unbiased estimates in further analyses. Thus, the present sample may be considered statistically representative of the target Italian employed working population in 2013.

### 2.3. Measures

The standardized questionnaire developed to conduct the CATI interviews in the INSuLa project was based on the findings of a literature review and a benchmark analysis of the most prominent European surveys and tools in the field (for details, see [80]). The measure of psychosocial risk employed in this study was comprised of 7 items evaluating different psychosocial conditions regarding the organization of work and the social context, selected from the Management Standards Indicator Tool (MS-IT, [81]; for the Italian validation, see [82,83]). Selected items were “I have unachievable deadlines” (demands); “I have a choice in deciding what I do at work” (control); “I get help and support I need from colleagues” (peer support); “I can talk to my line manager about something that has upset or annoyed me about work” (managerial support); “I am clear about the goals and objectives for my department” (role); “I am subject to bullying at work” (relationships); and “I have sufficient opportunities to question managers about change at work” (change). Items were endorsed on a 5-point Likert-type scale (ranging from 1 = strongly disagree to 5 = strongly agree). For sake of clarity, “positively” worded items (i.e., control, peer support, managerial support, role, and change items) were reverse coded in order to reflect a “lack of” those characteristics. Accordingly, a higher total score of the scale reflects higher levels of psychosocial risk.

### 2.4. Analytic Approach

First, we classified employees into three distinct age classes: younger, middle-aged, and older employees. As noted by Costanza, Badger, Fraser, Severt, and Gade [84], there is no convergence around a common operational definition of what a young, middle-aged, and old employee is. Accordingly, we used four different criteria to generate such groups in order to avoid capitalization on chance of our study findings. The first criterion (generational) was used to distinguish the employees in terms of their generational membership. Consistent with Lyons and Kuron [85], we distinguished younger, middle-aged, and older workers, respectively, as “Generation Y” (or Millennial Generation, born between 1981 and 1996, 19–32 years old at the time of survey), “Generation X” (born between 1965 and 1980, 33–48 years old), and “Baby Boomers” (born between 1946 and 1964, 49–64 years old). The second criterion (lifespan) allowed distinguishing employees’ age classes on the basis of three general stages of human development across the lifespan. Consistent with Merriam [86], we therefore distinguished these age groups in the following categories: young adulthood (19–35 years old), middle age (36–49 years old), and pre-retirement life periods (50 or more years old). A third criterion (career stages) was derived from Super’s theory [87] of career stages (see also [88]). In this case, younger, middle-aged, and older employees were identified on the basis of the trial (19–30 years old), stabilization (31–44 years old), and maintenance (45 or more years old) career stages. Finally, we also used a purely data driven criterion (empirical) by splitting the frequency distribution continuous age variable into tertiles; in this case, the age of younger employees ranged from 19 to 33 years old, middle-aged from 34 to 49 years old, and older ones were50 or more years old. In line with our hypotheses, we thus used each of these criteria to create an age class*employment status classification which gave rise to six final groups of employees (i.e., younger contingent, younger permanent, middle-aged contingent, middle-aged permanent, older contingent, and older permanent employees). Further analyses were conducted and replicated on each age*employment status classification.

As argued by Edwards et al. [81], the seven dimensions of the Management Standard Indicator Tool are subsumed under a common higher-order factor. Moreover, recent empirical evidences suggest that these dimensions are strongly correlated [83]. Thus, we evaluated the measurement invariance [89] of a confirmatory factor model positing a single latent variable loaded by the seven indicators of the study measure across the age*employment status groups. In this case, the evaluation of measurement invariance allows for ascertaining that the 7-item measure has consistent psychometric properties across groups and to compare their means at the latent level (i.e., partialled out from measurement error). Overall model fit was assessed with commonly used indices (see [90]) and any worsening of model fit between adjacent models (e.g., metric vs. configural invariance) was evaluated with the difference in comparative fit index criterion (∆CFI). If ∆CFI > 0.01, the more restrictive model (e.g., scalar vs. metric invariance) should be rejected [91]. In such cases, modification indices were inspected and equality constraints (one by one) were released accordingly (see [92]) until the ∆CFI criterion was satisfied. Note that in cases of partial scalar invariance, latent mean comparison among groups is still meaningful [93]. Finally, the equality between latent means from different groups was tested with the Wald χ² test. In this context, the interpretation of the Wald χ² is similar to the “omnibus” F test in the ANOVA analytical framework (see [94]), since it evaluates the null hypothesis of no difference between groups’ latent means. Factor scores were calculated from the most parsimonious model and used as the dependent variable for the informative hypothesis testing purposes. All multi-group models were estimated with sampling weights (see [95]) in order to obtain unbiased estimates consistent with the original sampling plan [96]. 

Since the items of our study measure are empirically congeneric (see [97]), we avoided relying on Cronbach’s alpha coefficients to evaluate the internal consistency among the items. Therefore, reliability of the latent dimension was evaluated for all groups by composite (ω) and maximal reliability (*H*) indices. These model-based reliability coefficients were calculated relying on the standardized estimates from the most restrictive invariance model. Similarly to the interpretations of Cronbach’s alpha coefficient, values higher than 0.70 are indicative of satisfying reliability [98].

Competing informative hypotheses were then tested and compared with the software BIEMS [99]. Since age effects may be conflated with those related to job tenure [100], all informative hypotheses were tested controlling for the potential effects of this latter variable. Specifically, job tenure was added as a covariate when testing group differences for all informative hypothesis models. For this purpose, default prior information was specified for all parameters (i.e., conjugate expected-constrained posterior prior, CECPP) while the covariate was previously standardized on the whole sample (for technical details, see [99]).

The evidence of each informative hypothesis was expressed by the relative Bayes factor (BF) and the posterior model probability (PMP).The BF quantifies the evidence of each informative hypothesis with respect to an unconstrained hypothesis (H_unc_) where no (in)equality constraints are placed between group means. A BF > 20 can be interpreted as a sign of strong support from the data to the informative hypothesis [74] (p. 83). The ratio among BFs of two different informative hypotheses (e.g., H_2_ vs. H_3_, that is BF_2,3_) provides the relative evidence of one informative hypothesis over the other. If this ratio is >1, one can conclude that the first hypothesis should be preferred [74]. Finally, the PMP is a standardized version of a single BF by the sum of all BFs, and it expresses proportionally the direct support from the data to a given hypothesis over the others considered simultaneously. 

Finally, standardized Cohen’s d latent coefficients [101] were computed as effect sizes of the differences among latent means and these coefficients were partialled out from potential confounding effects of job tenure by regressing the latent scoresin psychosocial risk on this control variable. Since generally accepted cut-offs for evaluating effect sizes (e.g., Cohen’s benchmarks, [102]) have been severely questioned (see [103]), we relied on the most up-to-date criteria provided by Paterson, Harms, Steel, and Credé [104].

## 3. Results

### 3.1. Descriptive Statistics

Table 2 provides descriptive statistics of the items in our study measure. As can be noted, the relationships item was severely and positively skewed, since the majority of people endorsed this item using low response categories. A similar situation was found for the demands item, although the impact of the skewness on frequency distribution was much less pronounced. For this reason, all multi-group confirmatory factor models (MG-CFA) were examined with the maximum likelihood estimation with robust standard errors and a mean- and variance-adjusted test statistic (MLMV) with listwise deletion to handle missing data. Since no missing data points in item responses were detected, the final sample remained unaltered.

Table 3 provides information about the multiple solutions adopted to derive age class*employment status groups. Polychoric correlations among the four different age group classifications ranged between 0.77 and 0.88 (mean = 0.82, SD = 0.05), suggesting that such classifications are not perfectly overlapping, and they might provide unique patterns of results in relation to the informative hypothesis testing. As can be observed, distribution of employees in young, middle-age, and old age categories (and, consequently, within each age class between permanent and contingent categories) issensitive to the selected criterion to derive age groups (except in the case of older employees for the generational and empirical criteria, which resulted in exactly the same number of employees both in the permanent and contingent categories). It is worth noting that cross-tabulations of age class with employment status revealed (regardless of the age splitting criterion used) a significant overrepresentation of younger employees within the contingent category, who were also underrepresented within the permanent category. The opposite pattern was found in all cases for older workers. These results were consistent with the more general picture of the actual employment distribution in Italy [105], which is in line with the overall European context [106].

### 3.2. Measurement Invariance Testing

Table 4 shows results from the measurement invariance testing. As can be noted, these analyses yielded exactly the same results regardless of the criterion used for distinguishing age classes. Specifically, metric invariance was fully reached, while the full scalar invariant condition was not supported. After inspecting modification indices, the intercept of the control item was released across groups and scalar invariance was partially reached. Finally, full strict invariance was tenable. Overall, these results allow for the meaningful comparison of group means at the latent level [107]. This result allowed comparing latent scale scores between groups partialled out from their measurement error.

Table 5 shows factor loadings and reliability estimates for all groups derived with different age-related splitting criteria. As can be noted, demands and relationships items loaded poorly in all groups regardless of the age splitting criteria (especially the first one). Informative analysis testing was also repeated considering factor scores calculated after excluding these two items from the most restrictive multi-group factor models, which found very similar results to what was observed without removing them. Thus, they were retained for further analytic purposes. This phenomenon is mainly due to the fact that they were the only negatively worded items of the entire set (see [108]). However, since their factor loadings were both statistically different from zero and in the expected direction, they were not excluded from further analytic purposes. Finally, factor scores were calculated from the most restrictive model (i.e., full metric, partial scalar, and full strict invariance model), representing the dependent variable of informative hypothesis testing.

### 3.3. Tests of Competing Informative Hypotheses

Table 6 presents results from the Bayesian evaluation of the competitive informative hypotheses. As can be noted, the two informative hypotheses mainly supported from the data were H_1_ (i.e., older employees are more at risk for in psychosocial factors for work-related stress) and H_9_ (i.e., older contingent employees are more at risk than their permanent colleagues which, in turn, are more at risk than all other employees). However, BFs and PMPs were higher for H_9_ than H_1_ in all cases and regardless of age group classification. Specifically, BF_9,1_ suggests that H_9_ was 1.45 times more likely than H_1_ after seeing the data if the generational criterion to derive age classes is applied while, respectively, it is 5.02, 1.64, and 1.90 more likely if the lifespan, career stages, and empirical criteria are applied. Overall, we can conclude that H_9_ informative hypothesis is the most likely among those tested on the study sample.

Table 7 presents the effect sizes related to group differences as devised by H_9_. In all cases and age classifications, such effects were statistically different from zero for *p* < 0.001. The standardized latent Cohen’s d related to the difference between older contingent and older permanent psychosocial risk ranged between 0.12 and 0.19 depending on age class criterion, while the distance between older permanent and other employees ranged between 0.08 and 0.09. Finally, the difference between average factor scores of older contingent and other employees ranged between 0.18 and 0.28. Consistent with guidelines provided by Paterson et al. [104], we can interpret the magnitude of effect sizes related to μOc > μOp as medium-low, and those concerning μOp > other employees and μOc > other employees, respectively, as low and medium.Although these effect sizes are far from high, it should be noted that they were estimated within a latent variable framework (i.e., they are partialled out from random measurement error) while controlling for job tenure effects. In other words, we ensured that such estimates were attributable only to meaningful group differences.

## 4. Discussion

Using a representative sample of the Italian workforce, the present study aimed to disentangle the interplay among age and employment status in shaping perceptions of psychosocial risk factors at work. Consistent with this, we reviewed theoretical approaches and empirical evidences linking both these objective features of the employee profile and we framed their interactive role in shaping perceptions of psychosocial risk factors within the COR theory [109]. Accordingly, while older employees may have depleted some key personal resources (e.g., cognitive abilities and physical skills) to handle with multiple job demands (e.g., task demands and lack of social support), being a contingent worker exposes the individual to a prolonged state of threat of loss of other important resources (e.g., economic stability) and to a set of specific strains (i.e., employment strains, see [72,73]). Thus, we expected older contingent workers would more negatively characterize their overall working conditions than older permanent ones, who, in turn, were expected to be more at risk than all other employees in the sample.

Specifically, our results are consistent with the primacy of loss and the loss spiral principles of the COR theory. While the first states that loosing key resources is more salient that gaining new ones, the second represents those situations in which the accumulation of losses in multiple resources may increase the likelihood of eroding other resources. In particular, our results suggest that aging processes combined with a contingent employment status may correspond to a higher vulnerability to negative work environments.

Although we formulated a specific hypothesis (H_9_) explicitly relying on COR theory predictions, our literature review regarding age effects on psychosocial risk factors at work depicted a fragmented scenario. Indeed, we found prior theoretical and empirical support for three different patterns of relationship among age and psychosocial risk factors: (1) older employees may experience lower levels of psychosocial stressors because they have accumulated more resources to offset their negative consequences; (2) older employees may experience such stressors with higher intensity because they have lost or depleted other key resources which are pivotal to hinder negative effects of high job demands (e.g., cognitive abilities and physical skills); and (3) middle-aged employees are more at risk than younger and older colleagues because they are invested with a higher volume of responsibilities and duties, both within and outside the workplace. Moreover, although being a contingent worker is commonly acknowledged as a risk factor potentially accruing the experience of poor working conditions, there is also some evidence that being a permanent employee may expose the individual to role-based overload [79].

The data supported our expectations over the other competitive hypotheses we formulated. Specifically, our substantive hypothesis H_9_ was much more strongly supported than the unconstrained hypothesis (H_unc_) and the “null” hypothesis (H_0_). Moreover, H_9_ was distinguishable from the second most informative competitive hypothesis (H_1_). Given these results, we concluded that older workers with contingent employment status were the most vulnerable category to psychosocial risks, followed by their permanent colleagues who, in turn, were more at risk than other employees of different ages regardless of their employment status. Importantly, these effects were detected across different age*employment status classifications after controlling for job tenure.

In line with this, and since the analysis of the effect sizes highlighted that especially older workers with contingent contractual arrangements may be more vulnerable to unfavorable psychosocial working conditions than others, we focused the discussion of practical implications of our findings on this specific group of employees.

### 4.1. Theoretical Implications

From a theoretical point of view, the present findings contribute to the extant literature in different ways. First, our results were fully consistent with predictions drawn from the COR theory [6,109]. In this sense, COR theory emphasizes the role of objective conditions that may be conducive to the development of strain and stress. Both age and employment status represent two objective characteristics of the general employee profile. Although their measurement is independent from psychological processes (on this point, see [110]), both of them subsume important normative individual differences for work-related stress processes. On the one hand, older workers may have lost or depleted key resources in coping with multiple demands that occur simultaneously in the workplace. In this regard, the SOC model applied to organizational contexts (see [111]) argued that the adoption of selective strategies concerned with aging processes within the workplace may stem from two different motives. 

Specifically, older employees may adopt specific strategies to reach their valued goals when they deliberately decide to select them, while they adopt loss-based selective strategies when they have to change their own goals because they have lost some relevant means to challenge them (e.g., cognitive resources). Since work goals are mainly set from top and middle management on the basis of stakeholders’ requirements (e.g., market demands), employees typically have limited autonomy to choose specific goals to pursue. Therefore, they may be more prone to adopt loss-based selective strategies to fit their actual resources into multiple work goals, and the mismatch between multiple goals and the limited resources they may offset can lead to perceiving one’s working conditions as worse than other employees. Moreover, being in a contingent status may exacerbate such a relationship. Although contingent employees may not necessarily experience higher subjective job insecurity than permanent ones (see [112]), they are exposed to unique sources of stress [66], both in and outside the workplace (see [72,73]). For this reason, being an older contingent employee may result in an accumulation of losses and threat for losses in key resources, resulting in a resource loss cycle (see [6]) that puts the older employee in a condition of higher vulnerability to psychosocial risk factors and their consequences, which generally turn into work-related stress [18].

Second, findings from the present study represent an interesting proof of integration between COR theory principles and transactional stress models (e.g., [113]). Although Hofboll et al. [6] strongly emphasized the role of objective variables as core antecedents of strain and work-related stress, our results show that age and employment status may explain the subjective experience of working conditions. For example, transactional models of stress could incorporate objective features of the employee profile as shapers of primary appraisal of environmental stimuli, which might provide incremental validity above and beyond psychological and other subjective factors.

Finally, to the best of our knowledge there are no previous studies examining the role of job contract as a demanding stressor across different life and career stages in relation to psychosocial risk factors for work-related stress. Although previous research considered contingent employment status as an objective proxy of subjective job insecurity (e.g., [58]), our results clearly highlight that non-permanent contracts are associated with a higher vulnerability to negative psychosocial working conditions only for older workers. In this sense, it would be of paramount importance to clarify the relationship between non-permanent contractual arrangements and subjective job insecurity in light of different life and career stages.

### 4.2. Practical Implications

Our findings identified older contingent employees as the subgroup most vulnerable to psychosocial risks, followed by their permanent peer counterparts. This reinforces the importance of efforts on the part of policy makers to avoid age discrimination within the workplace (e.g., [114]) and to provide financial support to contingent employees after job loss. However, being an older age contingent employee may have additional undesirable consequences that cannot be observed when separately examining ageing processes and employment status. For example, Wanberg, Kanfer, Hamann, and Zhang [115] found that older employees that lost their jobs experience more difficulties finding a new job than others, and their speed of reemployment is consistently slower. For these reasons, policy makers should protect thiscategory of employees from experiencing additional employment strains ([72,73]; see also [116]), which may add uncertainty to their already vulnerable condition.

Employers and managers may benefit from the present findings by considering possible job design interventions (e.g., [117]). For example, older contingent employees may be assigned work goals enhancing their improved resources (e.g., expertise and coping skills) rather than imposing excessive job demands (e.g., workload and time pressure) that require resources which progressively diminish during aging. In terms of job design, possible interventions should be developed by increasing autonomy and skill variety for old workers, considering that motivation is a key factor of age-related changes (e.g., [118,119]), and empirical evidence also suggests that frustration of psychological needs, such as motivational mechanisms, may explain the link between job insecurity and impaired well-being [120]. More work-life supportive policies could also be improved for older workers, such as telecommuting, flexible work arrangements or part-time work [121,122,123], in order to support their work–life balance and to face eldercare needs which represent a specific challenge in aging. In terms of health promotion, further research also suggests specific interventions to increase physical and intellectual activity in order to support physical and mental needs of older people [124,125]. Our results also suggest that good training practices for older workers should include working in smaller groups, giving additional time, and improving goal orientation skills (e.g., [126,127,128]), which could be more vulnerable in contingent working conditions. Moreover, managers and colleagues should avoid stigmatizing older contingent employees as a “peripheral” component of the organization by actively including them in organizational communications and involving them in relevant social exchange processes [129].

Finally, since older contingent (and older permanent) employees may “objectively” have fewer resources to counteract negative effects of their working conditions, their younger (and middle-aged) colleagues may offer their support in order to compensate for this shortage. In turn, older employees may reward their younger colleagues by offering their expertise in order to compensate for younger employees’ lack of other pivotal resources. Overall, organizations may effectively use reverse mentoring leverages [130,131] for bridging mutual gaps in important resources and skills among older and younger employees.

Finally, it should be noted that the global workforce is aging, and this phenomenon requires important economical investments for the implementation of sustainable health care and retirement systems [132,133]. Accordingly, employers and policy makers may develop appropriatestrategiesfor improving the successful adaptation of older contingent workers to retirement, especially in light of recent findings demonstrating that problematic retirement may lead to impaired mental health [134]. Being aware of the vulnerability of this specific target of the workforce should be the starting point for implementing focused and tailored interventions aimed at promoting sustainable stages of transition between their job career and retirement.

### 4.3. Limitations and Future Directions

Although the present study adds a valuable contribution to the literature, further supported by the use of a representative sample of the Italian population, it is not immune from some important limitations. First, we used a measure of psychosocial risk factors comprising only one item per the intended first-order latent dimensions (e.g., a single item to assess the control dimension). Moreover, two of these indicators reflected poorly the posited general latent dimension of psychosocial risks. While these items did not affect our substantive conclusions, future studies should replicate and extend our findings with more comprehensive measures of psychosocial risk factors using multiple items per factor and considering also other complementary instruments assessing psychosocial risk factors at work (e.g., the Copenhagen Psychosocial Questionnaire II, see [135]).

Second, the nature of our data was cross-sectional and based on self-reported information from single informants nested within a single national context. Although employees were surveyed with a rigorous procedure based on a nationally representative sampling strategy, both conditions limit the possibility to infer any causal relationship among our study variables and to generalize cross-culturally these results to other workforces. Future studies should replicate these findings using time spanned measurement occasions to measure independent and dependent variables by using data from different informants (e.g., employees and employers) in other countries.Moreover, futureresearch should consider further “objective” characteristics of employee profile that may contribute toexplain primary appraisals of psychosocial risk factors at work (e.g., average work hours per week).

Third, further research should investigate whether different age*employment status combinations are associated with relevant work-related stress outcomes (e.g., burnout) and if the impact of psychosocial risk factors on both strains and actual work-related stress may vary accordingly.

## 5. Conclusions

As we reviewed in the present paper, the impact of age and employment status on subjective perceptions (and their interplay) provided mixed findings. The present study formulated and tested multiple and competitive theoretically grounded hypotheses regarding the interplay of two objective characteristics of the employee profile (i.e., age and employment status) in shaping primary appraisals of psychological working conditions. In line with COR theory predictions, older workers in a contingent employment status were found to be the most vulnerable category, followed by their age peers with permanent contractual arrangements who, in turn, were more at risk than middle-aged and younger workersregardless of their work contracts. The present findings highlight the importance for policy makers and employers to carefully consider older workers (especially those in a contingent status) as more vulnerablethan others to a negative psychosocial work environment, and they may benefit from these findings by promoting effective compensatory strategies to bridge such disadvantage, such as job design interventions to reduce the impact of negative working conditions on older employees (especially those in contingent employment status) and reverse mentoring programs involving younger employees aimed at bridging mutual gaps.

## Figures and Tables

**Table 1:** Socio-demographic and occupational characteristics of the study sample (*n* = 8000).

	Male (*n* = 4314, 54%)		Femal (*n* = 3686, 46%)		Tota (*n* = 8000)	
	*n*	%	*n*	%	*n*	%
**Age**						
16–24	243	4.9%	182	4.5%	425	5.3%
25–34	897	20%	752	18.5%	1649	20.6%
35–44	1328	31.8%	1163	32.4%	2491	31.1%
45–54	1256	29.6%	1105	30.8%	2361	29.5%
55–64	581	13.7%	493	13.8%	1074	13.4%
Missing	0	0%	0	0%	0	0%
**Organizational tenure**						
Less than one year	288	6.5%	249	6.5%	537	6.7%
1–5 years	882	20.4%	827	21.9%	1709	21.4%
6–10 years	871	20.6%	717	19.1%	1588	19.9%
11–15 years	670	15.7%	604	16.7%	1274	15.9%
Over 15 years	1594	36.9%	1297	35.7%	2891	36.1%
Missing	0	0%	1	0%	1	0%
**Firm size**						
1–9 employees	614	14.3%	637	17.2%	1251	15.6%
10–49 employees	868	20.2%	698	18.9%	1566	19.6%
50–249 employees	898	20.9%	816	22.1%	1714	21.4%
Over 250 employees	1779	41.3%	1358	36.8%	3137	39.2%
Missing	146	3.4%	186	5%	332	4.2%
**Macro-area**						
North	2236	51.9%	2064	55.9%	4300	53.8%
Center	873	20.3%	808	21.9%	1681	21%
South and Islands	1196	27.78%	823	22.3%	2019	25.2%
Missing	0	0%	0	0%	0	0%
**Nationality**						
Italian	4,191	97.4%	3586	97.1%	7777	97.2%
Foreign	114	2.6%	109	3%	223	2.8%
Missing	0	0%	0	0%	0	0%
**Economic activity (NACE Rev.1.1 - ATECO 2002)**						
Agriculture, hunting, fishing (A,B)	131	3%	55	1.5%	186	2.3%
Manufacturing/industry/energy (C,D,E)	1356	31.5%	500	13.5%	1856	23.2%
Construction (F)	409	9.5%	35	0.9%	444	5.6%
Wholesale and retail trade, hotels and restaurants (G,H)	694	16.1%	719	19.5%	1413	17.7%
Transport and storage and communication (I)	477	11.1%	157	4.3%	634	7.9%
Financial intermediation, real estate, renting and business activities (J,K)	422	9.8%	516	14%	938	11.7%
Health and social work (N)	194	4.5%	504	13.6%	698	8.7%
Education and public administration (M, L)	463	10.8%	730	19.8%	1193	14.9%
Other community and personal services (O,P,Q)	159	3.7%	479	13%	638	8%
Missing	0	0%	0	0%	0	0%

**Table 2:** Descriptive statistics for the items of the study measure.

	Mean	SD	Skewness	Kurtosis
it1 Demands: I have unachievable deadlines.	1.87	1.08	1.07	0.29
it2 Control: I have a choice in deciding what I do at work.^R^	2.63	1.15	0.27	−0.53
it3 Peer Support: I get help and support I need from colleagues.	2.43	1.04	0.32	−0.28
it4 Management Support: I can talk to my line manager about something that has upset or annoyed me about work.^R^	2.29	1.17	0.57	−0.49
it5 Role: I am clear about the goals and objectives for my department.^R^	2.18	1.05	0.56	−0.28
it6 Relationships: I am subject to bullying at work.	1.18	0.58	3.98	17.41
it7 Change: I have sufficient opportunities to question managers about change at work.	2.64	1.17	0.20	−0.72

Notes: SD = standard deviation. Scores of items marked with the superscript ^R^ were reverse coded.

**Table 3:** Alternative age class*employment status classifications used for the present study.

	Younger Employees		Middle-Aged Employees		Older Employees	
	Perm.	Cont.	Perm.	Cont.	Perm.	Cont.
Generational Criterion	780 (9.8%)	388 (3.9%)	3669 (46.1%)	583 (7.3%)	2349 (29.5%)	188 (2.4%)
Lifespan Criterion	1351 (17%)	533 (6.7%)	3327 (41.8%)	464 (5.8%)	2120 (26.6%)	162 (2%)
Career Stages Criterion	484 (6.1%)	329 (4.1%)	3117 (39.2%)	527 (6.6%)	3197 (40.2%)	303 (2.8%)
Empirical Criterion	2087 (26.2%)	641 (8%)	2362 (29.7%)	330 (4.1%)	2349 (29.5%)	188 (2.4%)

Notes: Generational criterion = younger employees are 19–32 years old, middle-aged are 33–48 years old, and older ones are 49 years old or older. Lifespan criterion = younger employees are 19–35 years old, middle-aged are 36–49 years old, and older ones are 50 years old or older. Career stages criterion = younger employees are 19–30 years old, middle-aged are 31–44 years old, and older ones are 45 years old or older. Empirical criterion = younger employees are 19–33 years old, middle-aged are 34–49 years old, and older ones are 50 years old or older.

**Table 4:** Measurement invariance of the single-factor model across age*employment status groups derived with different criteria for age classes.

	Tested Model	Model Comparison	χ²	*df*	RMSEA (90% CI)	CFI	TLI	SRMR	∆CFI
**Generational** **Criterion**	1. Configural	–	200.72 ***	84	0.032 (0.027–0.038)	0.982	0.973	0.023	–
	2. Metric	2 vs. 1	231.53 ***	114	0.028 (0.023–0.033)	0.982	0.980	0.030	0
	3. Scalar	3 vs. 2	340.83 ***	144	0.032 (0.028–0.037)	0.969	0.973	0.036	0.013
	4. Partial Scalar ^†^	4 vs. 2	285.95 ***	139	0.028 (0.024–0.033)	0.977	0.979	0.031	0.005
	5. Strict_(with partial scalar)_	5 vs. 4	301.96 ***	174	0.024 (0.019–0.028)	0.980	0.986	0.055	−0.005
**Lifespan** **Criterion**	1. Configural	–	210.35 ***	84	0.034 (0.028–0.039)	0.981	0.971	0.024	–
	2. Metric	2 vs. 1	232.86 ***	114	0.028 (0.023–0.033)	0.982	0.980	0.029	−0.001
	3. Scalar	3 vs. 2	348.30 ***	144	0.033 (0.028–0.037)	0.969	0.973	0.034	0.013
	4. Partial Scalar ^†^	4 vs. 2	288.60 ***	139	0.029 (0.024–0.033)	0.977	0.979	0.031	0.005
	5. Strict_(with partial scalar)_	5 vs. 4	288.51 ***	174	0.022 (0.018–0.027)	0.982	0.987	0.046	−0.005
**Career Stages** **Criterion**	1. Configural	–	201.33 ***	84	0.032 (0.027–0.038)	0.982	0.973	0.023	–
	2. Metric	2 vs. 1	231.29 ***	114	0.028 (0.023–0.033)	0.982	0.980	0.027	0
	3. Scalar	3 vs. 2	341.41 ***	144	0.032 (0.028–0.037)	0.970	0.974	0.034	0.012
	4. Partial Scalar	4 vs. 2	287.65 ***	139	0.028 (0.024–0.033)	0.977	0.979	0.031	0.005
	5. Strict_(with partial scalar)_	5 vs. 4	325.70 ***	174	0.026 (0.021–0.030)	0.977	0.983	0.072	0
**Empirical** **Criterion**	1. Configural	–	198.96 ***	84	0.032 (0.026–0.038)	0.983	0.974	0.023	–
	2. Metric	2 vs. 1	227.20 ***	114	0.027 (0.022–0.033)	0.983	0.981	0.029	0
	3. Scalar	3 vs. 2	341.60 ***	144	0.032 (0.028–0.037)	0.970	0.974	0.034	0.013
	4. Partial Scalar ^†^	4 vs. 2	284.63 ***	139	0.028 (0.023–0.033)	0.978	0.980	0.031	0.005
	5. Strict_(with partial scalar)_	5 vs. 4	266.86 ***	174	0.020 (0.015–0.025)	0.986	0.990	0.039	−0.008

Notes: int. = intercept; RMSEA = root mean square error of approximation; CFI = comparative fit index; TLI = Tucker–Lewis index or non-normed fit index; SRMR = standardized root mean squared residual. ^†^ In the partial scalar model, the control item intercept was released across all groups. *** *p* < 0.001

**Table 5:** Standardized factor loadings and reliability coefficients from the final multi-group models.

**Generational Criterion**		**Younger**		**Middle-Aged**		**Older**	
		**Perm.**	**Cont.**	**Perm.**	**Cont.**	**Perm.**	**Cont.**
	**it1**	0.16	0.16	0.17	0.16	0.17	0.16
	**it2**	0.55	0.54	0.56	0.54	0.57	0.56
	**it3**	0.50	0.49	0.51	0.49	0.52	0.51
	**it4**	0.70	0.69	0.71	0.69	0.72	0.71
	**it5**	0.59	0.58	0.60	0.58	0.61	0.60
	**it6**	0.29	0.28	0.30	0.28	0.30	0.30
	**it7**	0.72	0.72	0.74	0.72	0.74	0.73
	**ω**	0.71	0.70	0.72	0.70	0.73	0.72
	***H***	0.78	0.77	0.79	0.77	0.79	0.79
**Lifespan Criterion**		**Younger**		**Middle-Aged**		**Older**	
		**Perm.**	**Cont.**	**Perm.**	**Cont.**	**Perm.**	**Cont.**
	**it1**	0.16	0.15	0.16	0.16	0.17	0.17
	**it2**	0.56	0.53	0.56	0.54	0.57	0.56
	**it3**	0.51	0.49	0.51	0.50	0.53	0.52
	**it4**	0.71	0.69	0.71	0.69	0.72	0.72
	**it5**	0.60	0.58	0.60	0.58	0.61	0.61
	**it6**	0.30	0.28	0.30	0.28	0.31	0.30
	**it7**	0.73	0.71	0.74	0.72	0.74	0.74
	**ω**	0.72	0.70	0.72	0.70	0.73	0.73
	***H***	0.79	0.76	0.79	0.77	0.80	0.79
**Career Stages Criterion**		**Younger**		**Middle-Aged**		**Older**	
		**Perm.**	**Cont.**	**Perm.**	**Cont.**	**Perm.**	**Cont.**
	**it1**	0.15	0.15	0.17	0.16	0.17	0.16
	**it2**	0.52	0.52	0.56	0.54	0.56	0.56
	**it3**	0.48	0.48	0.52	0.50	0.52	0.51
	**it4**	0.68	0.68	0.72	0.70	0.72	0.71
	**it5**	0.56	0.56	0.61	0.58	0.61	0.60
	**it6**	0.27	0.27	0.30	0.29	0.30	0.30
	**it7**	0.70	0.70	0.74	0.72	0.74	0.73
	**ω**	0.70	0,7	0.73	0.71	0.73	0.72
	***H***	0.75	0.75	0.79	0.77	0.79	0.79
**Empirical Criterion**		**Younger**		**Middle-Aged**		**Older**	
		**Perm.**	**Cont.**	**Perm.**	**Cont.**	**Perm.**	**Cont.**
	**it1**	0.16	0.15	0.17	0.16	0.17	0.16
	**it2**	0.56	0.53	0.56	0.55	0.57	0.56
	**it3**	0.52	0.49	0.52	0.51	0.53	0.51
	**it4**	0.71	0.69	0.71	0.70	0.72	0.71
	**it5**	0.60	0.58	0.60	0.59	0.61	0.60
	**it6**	0.30	0.28	0.30	0.29	0.30	0.30
	**it7**	0.73	0.71	0.74	0.73	0.74	0.73
	**ω**	0.72	0.70	0.72	71	0.73	0.72
	***H***	0.79	0.76	0.79	0.78	0.79	0.79

Notes: All factor loadings are significant for *p* < 0.001. ω = composite reliability; *H* = maximal reliability.

**Table 6:** Bayesian evaluation of the study informative hypotheses.

	(In)equality Constraints	Generational		Lifespan		Career Stages		Empirical	
		Criterion		Criterion		Criterion		Criterion	
		BF	PMP	BF	PMP	BF	PMP	BF	PMP
**H_0_**	μY*_p_* = μY*_c_* = μM*_p_* = μM*_c_* = μO*_p_* = μO*_c_*	1.69	0.01	0.13	0.00	4.65	0.09	1.61	0.01
**H_1_**	μO*_p_* = μO*_c_* > μM*_p_* = μM*_c_* = μY*_p_* = μY*_c_*	**40.1**	**0.35**	**12.75**	**0.16**	**14.01**	**0.27**	**58.89**	**0.32**
**H_2_**	μM*_p_* = μM*_c_* > μY*_p_* = μY*_c_* = μO*_p_* = μO*_c_*	3.91	0.03	0.00	0.00	0.06	0.00	0.03	0.00
**H_3_**	μY*_p_* = μY*_c_* > μM*_p_* = μM*_c_* = μO*_p_* = μO*_c_*	0.02	0.00	0.00	0.00	0.09	0.00	0.03	0.00
**H_4_**	μY*_c_* = μM*_c_* = μO*_c_* > μY*_p_* = μM*_p_* = μO*_p_*	0.1	0.00	0.01	0.00	0.20	0.00	0.10	0.00
**H_5_**	μY*_p_* = μM*_p_* = μO*_p_* > μY*_c_* = μM*_c_* = μO*_c_*	0.32	0.00	0.02	0.00	0.61	0.01	0.4	0.00
**H_6_**	μO*_c_* > μO*_p_* = μM*_p_* = μM*_c_* = μY*_p_* = μY*_c_*	1.34	0.09	3.09	0.04	8.57	0.17	11.9	0.06
**H_7_**	μM*_c_* > μM*_p_* = μY*_p_* = μY*_c_* = μO*_p_* = μO*_c_*	0.12	0.00	0.00	0.00	0.18	0.00	0.08	0.00
**H_8_**	μY*_c_* > μY*_p_* = μM*_p_* = μM*_c_* = μO*_p_* = μO*_c_*	0.05	0.00	0.00	0.00	0.11	0.00	0.04	0.00
**H_9_**	μO*_c_* > μO*_p_* > μM*_p_* = μM*_c_* = μY*_p_* = μY*_c_*	**58.22**	**0.51**	**64.08**	**0.80**	**23.00**	**0.45**	**111.78**	**0.60**
**H_10_**	μM*_c_* > μM*_p_* > μY*_p_* = μY*_c_* = μO*_p_* = μO*_c_*	0.01	0.00	0.00	0.00	0.00	0.00	0.00	0.00
**H_11_**	μY*_c_* > μY*_p_* > μM*_p_* = μM*_c_* = μO*_p_* = μO*_c_*	0.01	0.00	0.00	0.00	0.00	0.00	0.00	0.00
**BF_9,1_**		1.45		5.02		1.64		1.90	

Notes: Each informative hypothesis was tested including standardized job tenure as a covariate. Y*_p_* = younger permanent group; Y*_c_* = young contingent group; M*_p_* = middle-aged permanent group; M*_c_* = middle-aged contingent group; O*_p_* = older permanent group; O*_c_* = older contingent group; BF = Bayes factor; PMP = posterior model probability; BF_9,1_ = informative evidence of H_9_ over H_1_. BFs and PMPs of the two most likely informative hypotheses are highlighted in bold.

**Table 7:** Effect sizes of group differences.

	μO*_c_* > μO*_p_*	μO*_p_* > Other Employees	μO*_c_* > Other Employees
**Generational Criterion**	0.16 (0.21–0.11)	0.08 (0.13–0.03)	0.23 (0.28–0.18)
**Lifespan Criterion**	0.19 (0.24–0.14)	0.09 (0.14–0.04)	0.28 (0.33–0.24)
**Career Stages Criterion**	0.12 (0.17–0.07)	0.09 (0.14–0.04)	0.18 (0.23–0.13)
**Empirical Criterion**	0.16 (0.21–0.11)	0.08 (0.12–0.03)	0.23 (0.28–0.19)

Notes: Effect sizes are standardized latent Cohen’s d and they were estimated controlling for job tenure, with their associated 99% confidence intervals reported in parentheses. O*_p_* = older permanent group; O*_c_* = older contingent group.
